# Authors’ Reply: Rhabdomyolysis-Induced Acute Kidney Injury in Austere Environments Highlights Need for Specific Treatment

**DOI:** 10.1016/j.ekir.2023.04.007

**Published:** 2023-04-14

**Authors:** Jessica F. Hebert, Michael P. Hutchens

**Affiliations:** 1 Department of Anesthesiology and Perioperative Medicine, Oregon Health & Science University, Portland, Oregon, USA; 2 Operative Care Division, Portland Veterans Affairs Medical Center, Portland, Oregon, USA

**The Author Replies:**

We would like to thank Drs. Govindan, Fernando, and Kurien for their interest in our review of the mechanisms and emerging therapies for rhabdomyolysis-induced acute kidney injury (RIAKI).^1^ Rhabdomyolysis has multiple etiologies, including crush injuryS1, overexertionS2, viral infection (including COVID-19)S3^,^S4, or drug useS5; however, any injury resulting in the destruction of skeletal muscle can be an attributable cause of RIAKI. We agree with their assertion that venom from multiple animal species, including snakes and wasps, can cause rhabdomyolysis and subsequent acute kidney injury (AKI).S6^,^S7 Because of the critical and urgent nature of the injury and frequency in austere medical environments such as rural areas, venom-induced RIAKI is an important etiology that should inform efforts to derive novel treatment.

The authors describe a 46-year-old male patient presenting with acute tubular injury secondary to leptospirosis infection, which has been previously linked to AKI in humans,^2^ and RIAKI in a guinea pig model.^3^ AKI associated with hypotension, was identified by elevated serum creatinine and renal biopsy before the etiology was confirmed. Tubular casts observed by immunohistochemistry and Masson’s trichrome are cited as principal observations for the resultant RIAKI. Their finding of tubular epithelial injury in Figure 1 is very interesting because, as our review discusses, muscle metabolites released in the bloodstream are readily filtered by the glomerulus and enter the proximal tubule. Lining the tubule are megalin-expressing epithelial cells which endocytose myoglobin.^4^^,^S8 Intracellularly, injury results from free iron-mediated production of reactive oxygen species.

This case of a reproductive-age male highlights essential facets of AKI care which may be underappreciated. AKI is frequently described as a disease of the aged with no treatment. However, in the case described, leptospirosis was identified as a cause of rhabdomyolysis; prospective or early identification of infectious or other known precipitants of rhabdomyolysis offers potential opportunity for treatment through inhibition of myoglobin uptake or cell injury, such as with cilastatin, a megalin inhibitor,S9 which is also thought to have broad AKI-protective properties.^4^^,^S10^,^S11 We are encouraged that increasing attention to RIAKI may improve early identification and increase the likelihood of successful adoption of specific treatment.

## Supplementary Material

Supplementary File (PDF)

**Supplementary References.**
