# Supplementary material for: Authors’ Reply: Rhabdomyolysis-Induced Acute Kidney Injury in Austere Environments Highlights Need for Specific Treatment
Source: Kidney Int Rep. 2023 Apr 14;8(6):1274–5. doi: 10.1016/j.ekir.2023.04.007 (PMC10239767; doi:10.1016/j.ekir.2023.04.007)
Supplement: Supplementary File (PDF) [file mmc1.pdf]

Supplemental References:

S1. Malinoski DJ, Slater MS, Mullins RJ. Crush injury and rhabdomyolysis. *Crit Care Clin* 2004; **20:** 171-192.

S2. Luetmer MT, Boettcher BJ, Franco JM*, et al.* Exertional Rhabdomyolysis: A Retrospective Population-based Study. *Med Sci Sports Exerc* 2020; **52:** 608-615.

S3. Fadila MF, Wool KJ. Rhabdomyolysis secondary to influenza a infection: a case report and review of the literature. *N Am J Med Sci* 2015; **7:** 122-124.

S4. Nugent J, Aklilu A, Yamamoto Y*, et al.* Assessment of Acute Kidney Injury and Longitudinal Kidney Function After Hospital Discharge Among Patients With and Without COVID-19. *JAMA Network Open* 2021; **4:** e211095-e211095.

S5. Melli G, Chaudhry V, Cornblath DR. Rhabdomyolysis: An evaluation of 475 hospitalized patients. *Medicine* 2005; **84:** 377--385.

S6. Zhang L, Yang Y, Tang Y*, et al.* Recovery from AKI Following Multiple Wasp Stings: A Case Series. *Clinical Journal of the American Society of Nephrology* 2013; **8:** 1850-1856.

S7. Nishimura H, Enokida H, Kawahira S*, et al.* Acute Kidney Injury and Rhabdomyolysis After Protobothrops flavoviridis Bite: A Retrospective Survey of 86 Patients in a Tertiary Care Center. *Am J Trop Med Hyg* 2016; **94:** 474-479.

S8. Gburek J, Birn H, Verroust PJ*, et al.* Renal uptake of myoglobin is mediated by the endocytic receptors megalin and cubilin. *American Journal of Physiology-Renal Physiology* 2003; **285:** F451--F458.

S9. Hori Y, Aoki N, Kuwahara S*, et al.* Megalin blockade with cilastatin suppresses drug-induced nephrotoxicity. *Journal of the American Society of Nephrology* 2017; **28:** 1783--1791.

S10. Camano S, Lazaro A, Moreno-Gordaliza E*, et al.* Cilastatin attenuates cisplatin-induced proximal tubular cell damage. *J Pharmacol Exp Ther* 2010; **334:** 419-429.
